# Transfusion-dependent low-risk myelodysplastic patients receiving deferasirox: Long-term follow-up

**DOI:** 10.3892/ol.2013.1617

**Published:** 2013-10-10

**Authors:** SALVATORE IMPROTA, MARIA ROSARIA VILLA, ANTONIO VOLPE, ANGELA LOMBARDI, PAOLA STIUSO, NICOLA CANTORE, LUCIA MASTRULLO

**Affiliations:** 1 Hematology Division, P.O. San Gennaro ASL NA1 Centro, Naples I-80136, Italy; 2 Hematology Division, A.O.R.N. San G. Moscati, Avellino I-83100, Italy; 3 Department of Biochemistry, Biophysics and General Pathology, Second University of Naples, Naples I-80138, Italy

**Keywords:** myelodysplastic syndromes, transfusion dependence, iron overload, chelation, deferasirox, erythroid response

## Abstract

Myelodysplastic syndromes (MDSs) are characterized by ineffective hematopoiesis that results in peripheral cytopenias. Anemia is the most common symptom of MDS and the majority of patients become transfusion-dependent with the risk of iron overload, which may lead to cardiac, hepatic and endocrine complications. Deferasirox is an orally available iron chelator administered once-daily in transfusion-dependent patients with various chronic anemias. Its efficacy has been established in controlled clinical trials. In the present study, we describe our experience with 55 consecutive MDS patients [International Prognostic Scoring System risk score of low (n=32) or intermediate-1 (n=23)] treated with deferasirox in a routine clinical setting following Consensus Guidelines on Iron Chelation Therapy. According to WHO classifications, patients had refractory anemia (n=30), refractory anemia with ringed sideroblasts (n=16), refractory cytopenia with multilineage dysplasia (n=8) or refractory cytopenia with multilineage dysplasia and ringed sideroblasts (n=1). The median monthly transfusion requirement at baseline was 3 units. Patients received a starting dosage of 10 mg/kg/day, subsequently titrated according to serum ferritin (SF) levels which were measured monthly. Safety assessment included monitoring of liver and renal parameters and recording adverse events (AE) during treatment. At the baseline, the mean ± SD SF level was 2,362±172 ng/ml and after 24 months, the mean ± SD decrease in SF was 1,679±209 ng/ml. Sixteen patients had sustained hematological improvement meeting International Working Group 2006 criteria. One patient became transfusion-independent. No severe AE were reported. In conclusion, deferasirox therapy was effective and safe in reducing transfusional iron overload and it reduces transfusion requirement in a subset of patients.

## Introduction

Myelodysplastic syndromes (MDSs) are clonal hematopoietic stem cell disorders characterized by ineffective dysplastic hematopoiesis involving one or more cell lineages, and by peripheral-blood cytopenias with a high risk of progression to acute myeloid leukemia (AML) (1). MDS is most prevalent in white males and the incidence increases markedly with age. The documented disease burden is expected to increase in the near future, due to an aging population and improving awareness of the disease. Approved therapies, including lenalidomide, azacitidine and decitabine are now available for patients who are ineligible for potentially curative hematopoietic stem cell transplantation. These achieve hematological improvement and enhance the quality of life of patients who previously would have received supportive care alone. Thus, the mainstay of treatment for patients with MDS is supportive red blood cell (RBC) transfusions. Repeated transfusions eventually lead to iron overload with an increased risk of associated comorbidity and mortality that is independent of the underlying hematological disease (2). Retrospective studies have revealed that iron toxicity associated with transfusion burden is associated with reduced survival in patients with MDS (3,4). This is particularly problematic in patients with lower risk MDS due to the longer life expectancy.

The most common non-leukemia-related causes of mortality are cardiac failure (51%) and infections (31%) (3). Infections may be due to underlying neutropenia or to transfusional iron overload (5). There is growing evidence that adequate iron chelation therapy improves survival in International Prognostic Scoring System (IPSS) lower-risk MDS patients with iron overload and may delay AML transformation (1,6–9). Guidelines recommend that iron overload be managed with chelation therapy (10).

Until recently, desferoxamine and deferiprone were the only drugs available for the treatment of transfusional iron overload. However, neither provides satisfactory chelation therapy for controlling iron toxicity. According to the pharmacokinetic properties of desferoxamine, in order to be effective, it must be administered as a slow infusion over the course of 8–12 h and this must be repeated 5–7 days/week. This regimen is contraindicated in patients with thrombocytopenia and the inconvenience often results in low compliance (11). Deferiprone is not approved for MDS and is not recommended, as it causes neutropenia and agranulocytosis (12).

Deferasirox is a once-daily orally administered iron chelator approved for the treatment of iron overload in patients with transfusion-dependent anemias. It is efficacious and has an acceptable safety profile in adult and pediatric patients with transfusion-dependent thalassemia major and by various other chronic anemias (13). Recent guidelines from the Italian Society of Haematology recommend iron chelation with deferasirox for the treatment of low or intermediate-1 IPSS risk patients with MDS after they have received ≥20 units of packed RBCs (10). The initial dose of 10 mg/kg may be increased to 20–30 mg/kg based on iron transfusion load, serum ferritin (SF) levels and organ damage due to iron overload. In addition to reducing markers of iron overload, including SF, a number of recently published studies have reported improvements in hematological parameters and transfusion requirements in a portion of patients receiving iron chelation therapy with deferasirox (14–16, reviewed in 17). However, experience outside of clinical trials is lacking. We have investigated the safety and effectiveness of deferasirox therapy in reducing iron overload and transfusion requirements in a non-selected population of polytransfused low-risk MDS patients in a routine clinical setting.

## Patients and methods

Patients affected by low-risk MDS who had been transfusion-dependent for ≥1 year, had SF levels ≥2,000 ng/ml before starting iron chelation therapy and required ≥1 unit of RBCs/month to maintain Hb levels ≥8 g/dl were enrolled. Thus, these patients were eligible for analysis according to International Working Group 2006 criteria (18). The study was approved by the ethics committee of San Gennaro Hospital (Naples, Italy) and written informed consent was obtained from the patients or the patient’s family. Patients were treated at the San Gennaro Hospital (Naples, Italy) or the San G. Moscati Hospital (Avellino, Italy) between June 2006 and June 2012. All patients had low-risk disease, with IPSS scores of low or intermediate-1, and had been receiving only best supportive care.

Patients were treated according to the standard procedures at the enrolling centers. Initially, patients received deferasirox (10 mg/kg) orally once daily, as recommended by the Italian Society of Haematology, Italian Society of Sperimental Haematology and Italian Group for Bone Marrow Transplantation, Haematopoietic Stem Cells and Cell Therapy guidelines (10). Subsequent dosage adjustments in increments of 10 mg/kg/day were based on efficacy in terms of reduction in SF and safety parameters. The maximum dosage was 30 mg/kg/day (Table I).

Patients had an initial visit at baseline and follow-up visits at ~2-week intervals thereafter. At each visit, the deferasirox dosage, number of blood transfusions received, and changes in concomitant medications and iron intake were recorded. Hematological response was determined according to International Working Group 2006 criteria (18). Laboratory examinations for renal function (serum creatinine, 24-h creatinine excretion and creatinine clearance using the Cockcroft-Gault and Modification of Diet in Renal Disease formulae) and hepatic function (serum transaminases, bilirubin, alkaline phosphatase and γ-glutamyl transpeptidase) were performed at baseline and then weekly for the first month of treatment, monthly for the next 5 months and bimonthly thereafter. Examination of the ocular fundus and audiometric tests were performed at baseline and then every 6 months.

## Results

We enrolled 55 consecutive unselected patients affected by low-risk MDS who had been transfusion-dependent for ≥1 year. All patients (33 males and 22 females; median age, 70 years) had SF levels ≥2,000 ng/ml before starting iron chelation and required ≥1 unit of RBCs/month to maintain Hb levels at ≥8 g/dl (Table II). Four of the 55 patients had received prior chelation therapy with desferoxamine, which had been discontinued due to renal toxicity. The median transfusion requirement before starting treatment was 3 units/month.

**Figure 1:**
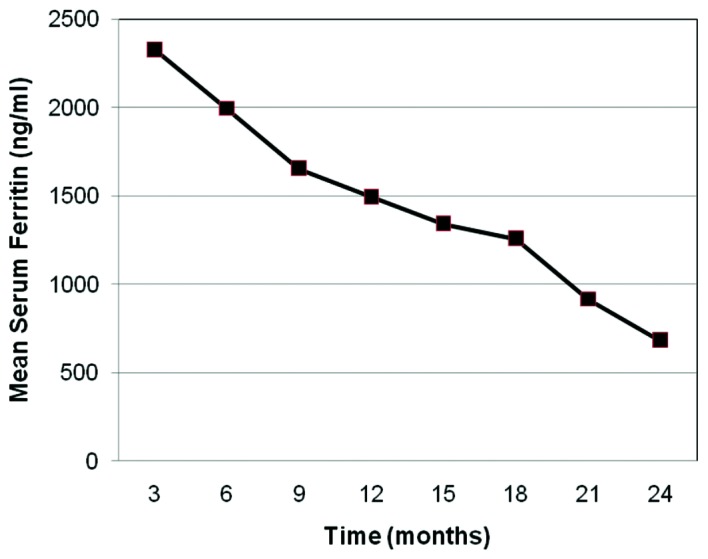
Mean serum ferritin levels (ng/ml) for 55 patients over the course of 24 months of treatment with deferasirox.

To date, all patients had >24 months of follow-up. Mean iron intake was 0.31 mg/kg/day at the baseline and 0.26 mg/kg/day after 24 months of therapy. All patients exhibited significant decreases in iron overload, as measured by SF, which were maintained after 2 years of therapy (mean absolute decrease in SF from baseline, 1,679±209 ng/ml; range 1,250–2,100 ng/ml), corresponding to a mean reduction of 71%. The rate of decrease in SF levels was relatively constant over the 24-month observation period (Fig. 1).

A sustained reduction in transfusion requirement was recorded in 16 of the 55 patients. Transfusion requirements were reduced by at least one transfusion per month after 6 months of therapy in all 16 patients who responded and were followed in all cases by further improvements at 24 months, meeting 2006 International Working Group criteria for hematological response (Table III). All 16 patients had erythroid responses, including one in a patient with multilinear dysplasia. There were no significant increases in neutrophil or platelet counts.

The mean monthly transfusion requirement at baseline was 3.25 units for the 16 patients who responded compared with 2.75 units among patients who did not have a hematological response; mean age was 69 years in the two groups. Among the patients who responded, less than one-fourth (3/16) were female, compared with approximately half of non-responders (19/39).

At baseline, these 16 patients had a mean transfusion requirement of 3.3 units/month, which decreased to 2.0 units/month after 6 months of treatment and to 1.1 units/month after 24 months. These values were relatively unchanged in the remaining patients, who required a mean 2.8, 2.7 and 2.8 units/month at baseline, 6 and 12 months, respectively. One patient became transfusion-independent after 13 months of iron chelation therapy. The patient was transfusion-free (SF, 800 ng/ml) with a hemoglobin level of ~8.8 g/dl in December 2012, 30 months after starting iron chelation therapy.

With regard to the onset of improvements in erythropoiesis, in 7/16 patients this occurred by 6 months, while all 16 patients had responses meeting International Working Group 2006 criteria after 12 months of treatment. Erythroid responses were observed when SF levels had been reduced by ~40% compared with baseline. There was a persistent reduction of transfusion requirements in approximately one third of patients.

Adverse events (AE) were documented in 34 patients (61.8%). All AEs were mild in severity (grade 1–2) and did not require discontinuation of therapy. The most common drug-related AEs were diarrhea, nausea, rash and headache (Table IV). There were no clinically significant alterations of renal or hepatic function. Similarly, optical and audiometric tests were normal.

## Discussion

Iron overload in patients with transfusion-dependent MDS is an important clinical problem, as secondary hemosiderosis may lead to organ failure in patients with a long life expectancy. Moreover, several studies have shown that the survival of patients with MDS is affected by transfusion dependence, which is a poor independent prognostic factor (19). Iron overload also has a negative impact on the success of stem cell transplantation (20).

In order to prevent iron toxicity, current guidelines indicate that iron chelation therapy should be considered in patients with SF levels of 1,000 ng/ml and who require 2 units RBC transfusions per month for ≥1 year.

Our findings are in agreement with results of the Evaluation of Patients’ Iron Chelation with Exjade^®^ (EPIC) study, the largest prospective evaluation of an iron chelation therapy conducted to date, which confirmed that deferasirox is an efficacious and generally well-tolerated treatment for iron overload in patients with transfusion-dependent disorders, including β-thalassemia and MDS (19). Our results, based on over 24 months of follow-up in non-selected MDS patients treated in a routine clinical setting, confirm the effectiveness and safety of deferasirox therapy in reducing the iron overload in polytransfused MDS patients.

We also identified a subset of 16 patients who had a reduction in transfusion requirement meeting International Working Group criteria for hematological response. This is in agreement with previous observations of hematological improvement with chelation therapy (reviewed in 17).

Moreover, a post hoc analysis of a subgroup of 341 patients with myelodysplastic syndromes enrolled in the EPIC study also identified hematological responses to deferasirox in a cohort of iron-overloaded patients (15). In this study, erythroid hematopoietic responses, according to International Working Group 2006 criteria, were observed in 53 of 247 patients eligible for analysis (21.5%) and there was a trend toward greater reductions in SF in patients with hematological responses. The erythroid response rate using the same criteria was similar in our study (29%, 16/55).

The results of the multicenter prospective Gimema trial of 152 consecutive patients (median age, 72 years) with low or intermediate-1 risk MDS were reported (16). After one year of deferasirox treatment, 22 patients had achieved transfusion independence, defined as no transfusion requirements for three consecutive months. The probability of acquiring transfusion independence after one year was 19.7% (95% CI, 19.4–20). While a similar percentage of our patients achieved a hematological response, only one patient achieved complete transfusion independence.

List *et al*(14) enrolled 176 patients with low or intermediate-1-risk MDS and SF levels >1,000 mg/l to receive treatment with deferasirox. The median reduction in SF was 36.7% in the 49 patients (28%) who completed 24 months of treatment. This is considerably less than our finding of a median reduction in SF of 70.2% at 24 months. Additionally, the rate of hematological erythroid responses defined by International Working Group 2006 criteria was lower in this study (30%, 51/176), compared with that in our patients (29%, 16/55).

However, the mechanism underlying the hematological response is not known. Reduction of oxidative stress caused by excess free iron in bone marrow has been implicated in bone marrow toxicity (21). Reduction of labile iron with chelation therapy has been proposed as the mechanism underlying hematological responses (17). Ghoti *et al*(22) measured labile iron (the redox-active form) and reactive oxygen species in blood cells from patients who had received deferasirox therapy for 3 months. The authors identified significant reductions in reactive oxygen species and lipid peroxidation, as well as an increase in the antioxidant, reduced glutathione.

A direct effect of deferasirox on hematopoiesis through the nuclear factor-κB (NF-κB) pathway has been proposed, based on *in vitro* experiments showing strong, iron-independent inhibition of NF-κB by deferasirox, but not other chelators (23). However, there is also limited evidence of hematological improvement in patients with MDS treated with desferoxamine (24), which argues against a direct role for deferasirox. Gattermann *et al*(15) found that, while treatment with deferasirox reduced labile iron to <0.4 μmol/l, there was no difference between hematological responders and non-responders. If removal of iron is labile, since it is the underlying mechanism of the hematological response, one may expect to see a difference between these groups.

The timing of hematological responses may give an indication of the mechanism. Seven of the patients in the present study had responded after 6 months of treatment and all 16 patients responded after 12 months. Patients appeared to respond when they had reached an ~40% reduction in SF. In the 3-year study by List *et al*(14), the median time to any type of hematological response was also ~6 months.

Further studies are warranted to define the role of deferasirox in reducing the blood transfusion request in a subset of patients affected by MDS that is refractory to other conventional therapies.

## Figures and Tables

**Table I:** Deferasirox dosage for all 55 consecutive patients.

Dosage	Patients (n=55)
Starting dose, mg/kg/day	10
Median daily deferasirox dose over the course of the study, mg/kg/day (range)	23 (10–30)
Dose adjustments for starting dose^a^, n (%)	
Unchanged	10 (18)
Increased	45 (82)
Dose adjustments during treatment^b^, n (%)	
Unchanged	38 (69)
Increased	11 (20)
Reduced	6 (11)

a Baseline compared with final visit;

b second month compared with final visit.

**Table II:** Demographic and clinical characteristics of 55 patients at diagnosis.

Characteristics	Patients
Male, n (%)	33 (60)
Female, n (%)	22 (40)
Median age, years (range, IQR)	70 (58–79, 9)
WHO classification, n	
RA	30
RARS	16
RCMD	8
RCMD-RS	1
Transfusion requirement, unit/month, mean ± SD	2.9±0.95
Prognosis (IPSS), n	
Low	32
Intermediate-1	23
Prognosis (WPSS), n	
Low	41
Intermediate-1	14
Serum ferritin, ng/ml, mean ± SD	2362±172
Hb, g/dl, mean ± SD	7.3±0.17

IQR, interquartile range; RA, refractory anemia; RARS, refractory anemia with ringed sideroblasts; RCMD, refractory cytopenia with multilineage dysplasia; RCMD-RS, refractory cytopenia with multilineage dysplasia and ringed sideroblasts; IPSS, International Prognostic Scoring System; WPSS, World Health Organisation Classification-Based Prognostic Scoring System; Hb, hemoglobin.

**Table III:** Characteristics of 16 patients meeting 2006 International Working Group criteria for haematological improvement while receiving deferasirox.

Gender	Age (years)	Histological classification	Prognosis (IPSS)	Transfusion support (units/month)			Serum ferritin (ng/ml)		Haemoglobin (g/dl)		
		
				Baseline	6 months	24 months	Baseline	24 months	Baseline	24 months	Change
M	72	RA	INT-1	3	2	1	2400	850	7.0	8.6	1.6
M	72	RARS	INT-1	3	2	1	2420	850	7.3	8.8	1.5
M	72	RA	INT-1	3	2	1	2440	850	7.3	8.8	1.5
M	60	RA	LOW	3	2	1	2350	750	7.3	8.9	1.6
M	73	RCMD	INT-1	3	2	1	2450	850	7.3	8.8	1.5
M	60	RA	LOW	3	2	1	2350	750	7.4	9.0	1.6
F	72	RA	LOW	3	2	1	2100	450	7.3	8.8	1.5
M	60	RA	LOW	3	2	1	2450	750	7.2	8.8	1.6
M	67	RARS	LOW	4	3	2	2540	700	7.0	8.6	1.6
M	67	RARS	LOW	4	3	2	2540	700	7.0	8.6	1.6
M	67	RARS	LOW	4	3	2	2540	700	7.0	8.6	1.6
M	74	RA	LOW	3	1	1	2350	800	7.2	8.7	1.5
F	74	RA	LOW	3	1	1	2350	800	7.4	9.1	1.7
M	74	RARS	INT-1	3	1	1	2350	800	7.0	8.6	1.6
F	67	RA	LOW	4	3	1	2340	700	7.0	8.5	1.5
M	74	RA	LOW	3	1	0	2350	800	7.0	8.8	1.8

IPSS, International Prognostic Scoring System; RA, refractory anemia; RARS, refractory anemia with ringed sideroblasts; RCMD, refractory cytopenia with multilineage dysplasia; INT-1, intermediate-1.

**Table IV:** Adverse events recorded in all 55 patients through 24 months of therapy with deferasirox. All adverse events were grade 1–2.

Adverse event	Events, n	Patients, n (%)
Diarrhea	20	15 (27.0)
Nausea	11	10 (18.0)
Rash	8	8 (14.5)
Headache	8	8 (14.5)
